# Temporary inactivation of the anterior part of the bed nucleus of the stria terminalis blocks alarm pheromone-induced defensive behavior in rats

**DOI:** 10.3389/fnins.2015.00321

**Published:** 2015-09-09

**Authors:** Tino Breitfeld, Johann E. A. Bruning, Hideaki Inagaki, Yukari Takeuchi, Yasushi Kiyokawa, Markus Fendt

**Affiliations:** ^1^Institute for Pharmacology and Toxicology, Otto-von-Guericke University MagdeburgMagdeburg, Germany; ^2^Laboratory of Veterinary Ethology, The University of TokyoTokyo, Japan; ^3^Center for Animal Research and Education, Nagoya UniversityNagoya, Japan; ^4^Center of Behavioral Brain Sciences, Otto-von-Guericke University MagdeburgMagdeburg, Germany

**Keywords:** anxiety, alarm pheromone, BNST, fear, muscimol, odor-induced anxiety, rats, risk assessment behavior

## Abstract

Rats emit an alarm pheromone in threatening situations. Exposure of rats to this alarm pheromone induces defensive behaviors, such as head out behavior, and increases c-Fos expression in brain areas involved in the mediation of defensive behaviors. One of these brain areas is the anterior bed nucleus of the stria terminalis (aBNST). The goal of the present study was to investigate if pharmacological inactivation of the aBNST by local microinjections of the GABA_A_ receptor-agonist muscimol modulates alarm pheromone-induced defensive behaviors. We first established the behavioral paradigm of alarm pheromone-induced defensive behaviors in Sprague-Dawley rats in our laboratory. In a second experiment, we inactivated the aBNST, then exposed rats to one of four different odors (neck odor, female urine, alarm pheromone, fox urine) and tested the effects of the aBNST inactivation on the behavior in response to these odors. Our data show that temporary inactivation of the aBNST blocked head out behavior in response to the alarm pheromone. This indicates that the aBNST plays an important role in the mediation of the alarm pheromone-induced defensive behavior in rats.

## Introduction

Pheromones are olfactory signals that are used for intraspecific communication (Karlson and Luscher, 1959). They can transmit different information, e.g., sex, age, and reproduction status, about the releaser (Beny and Kimchi, 2014). Additionally, pheromones have different ecological functions including marking a trail or territory, attracting potential mating partners, inducing aggregation or dispersion of conspecifics or warning conspecifics to potential danger (Hauser et al., 2011). Pheromones with the latter function are called alarm pheromones (Inagaki et al., 2014).

Alarm pheromones have been described in different mammalians including rats (Kiyokawa et al., 2006), mice (Brechbühl et al., 2013), deer (Müller-Schwarze et al., 1984), cattle (Boissy et al., 1998), pigs (Vieuille-Thomas and Signoret, 1992), and humans (Radulescu and Mujica-Parodi, 2013). For the alarm pheromone of rats, it is known that it is emitted from the perianal region (Kiyokawa et al., 2004), consists of at least two active ingredients, 4-methylpentanal and hexanal (Inagaki et al., 2014), and provokes in conspecifics a wide range of behavioral changes related to anxiety. For example, it aggravates stress-induced hyperthermia (Kikusui et al., 2001), enhances the acoustic startle reflex (Inagaki et al., 2009), and deteriorates male sexual behavior (Kobayashi et al., 2011). Alarm pheromone effects on defensive behavior can be tested in a modified open-field test paradigm where rats have a choice to stay in an open arena or escape into a safe hiding box. In this paradigm, exposure to alarm pheromone increases the time spent in the hiding box and induces typical “head out” behavior from the hiding box while the time spent in the open arena and for grooming is decreased (Kiyokawa et al., 2006).

In parallel with these behavioral analyses, the neural mechanisms underlying the alarm pheromone effects were also analyzed. The vomeronasal system was found to be involved in the detection of the alarm pheromone. Removal of the vomeronasal organ blocked the pheromone effects on stress-induced hyperthermia (Kiyokawa et al., 2007), acoustic startle reflex (Kiyokawa et al., 2013), and defensive behaviors in the modified open-field test (Kiyokawa et al., 2013). Mapping c-Fos expression throughout the brain in response to the alarm pheromone provided insights into the brain regions involved in pheromone effects (Kiyokawa et al., 2005b; Kobayashi et al., 2013, 2015). However, causal relationships between the alarm pheromone effects and any brain regions have not yet been demonstrated.

The bed nucleus of the stria terminalis (BNST) has been known as an important brain structure for the responses mediated by sustained fear or anxiety (Walker and Davis, 1997; Davis and Shi, 1999; Fendt et al., 2003; Takahashi et al., 2005; Poulin et al., 2009; Bota et al., 2012; Crestani et al., 2013). In addition, the BNST is one of the brain regions that compose the vomeronasal system (Brennan and Kendrick, 2006) and receives direct projection from the accessory olfactory bulb (AOB) (von Campenhausen and Mori, 2000), but not from the main olfactory bulb (Igarashi et al., 2012). Therefore, the BNST appears to be an excellent candidate for a brain relay structure connecting the vomeronasal system and alarm pheromone-induced behavioral changes. Indeed, increased c-Fos expression in response to the alarm pheromone or to its active ingredients has been repeatedly observed in the anterior part of the BNST (Kiyokawa et al., 2005b; Kobayashi et al., 2013, 2015; Inagaki et al., 2014).

The aim of the present study was to test the hypothesis that the BNST is involved in the mediation of alarm pheromone-induced behavioral changes. First, we established the modified open-field test paradigm (Kiyokawa et al., 2006) in our laboratory. In a second experiment, we assessed the role of the BNST, especially its anterior part (aBNST), in alarm pheromone-induced defensive behavior by temporally inactivating the aBNST by local injections of muscimol.

## Methods and materials

### Animals

All experiments were performed with naive male Sprague-Dawley rats (8–11 weeks at the start of the study). The animals were housed in groups of 4 to 6 in standard laboratory cages (standard conditions: 20–22°C; L 06:00; LD 12:12; humidity 50–65%). Food and water were available *ad libitum*. Some of the rats (15 male cagemates and 5 female littermates) were only used as donor animals for odor samples [alarm pheromone, neck odor, urine (urine from female rats)] but not for behavioral tests. All experiments were performed during the light phase. All experiments were performed according to international guidelines for ethical contact in the care and use of animals (2010/63/EU) and were approved by the local authorities (Landesverwaltungsamt Sachsen-Anhalt, Az. 42502-2-1238 UniMD).

### Preparation of odor samples

#### Alarm pheromone

We prepared a water solution containing alarm pheromone according to an established method that has been previously described in detail (Kiyokawa et al., 2004, 2005a). After anesthetizing the donor animal with pentobarbital sodium (50 mg/kg; i.p.), the anal region was cleaned and two intradermal needles (27G) were placed at the edge of both sides of the anal canal. The rat was put into an acrylic box (20 × 20 × 10 cm), without touching the walls. This box was previously washed with cleanser (7X, MP Biomedicals, Santa Ana, CA, USA) and the walls and the ceiling were sprayed with approximately 5 ml of purified water. Then, the box was closed and the needles were connected with a pulse generator (Model 2100, A-M Systems, Sequim, USA).

Afterwards, 15 electrical stimuli (10 mA, 1 s duration, 20 s inter-stimulus intervals) were applied to stimulate the perianal region of the donor rat. Subsequently, we waited for one more minute in order to let the released alarm pheromone dissolve in water droplets. The donor rat was then removed and the water droplets were collected and stored in a refrigerator until use (1–2 h later).

#### Water sample

Purified water was prepared before the experiment and used as a control water sample.

#### Fox urine

We used commercially available fox urine (Main Odor Solutions, Maine, USA).

#### Neck odor

For gaining the neck odor, which was used as a neutral odor stimulus, we used the same procedure as for gaining alarm pheromone. However, the two intradermal needles were placed in the neck.

#### Female rat urine

Female rats were placed in a metabolic cage (Tecniplast, Hohenpeißenberg, Germany) for 30 min and the urine delivered by these animals was collected. We collected several times from each animal and put all urine samples together, i.e., we had a mixture of urine from all different phases of the estrous cycles in the end.

Fox urine was used as an example odor from another species that is able to induce defensive behavior (Funk and Amir, 2000; Fendt, 2006; Wernecke et al., 2015). Neck odor and female urine were used as additional odors originating from the same species. Both odors should be neutral with respect to defensive behavior (Kiyokawa et al., 2004, 2005b). Female urine can also induces appetitive behaviors, however, this was not expected in the present study since sexually naive male rats and not freshly collected female urine from different estrous phases were used (cf. Lydell and Doty, 1972).

### Surgery

The animals were anesthetized with an isoflurane/oxygen mixture (5% isoflurane for induction, then 2.0–2.5%) and fixed into a stereotaxic apparatus. The skull was exposed and stainless steel guide cannulas (custom-made; diameter: 0.7 mm, length: 8.0 mm) were bilaterally implanted aiming at the aBNST: 0.1 mm caudal, ± 3.9 mm lateral, and 6.8 mm ventral to bregma at a 20° angle to avoid penetration of the ventricles. Cannulas were fixed with dental cement and anchoring screws. After the surgery, there was a recovery period of 5–8 days.

### Microinjections and drugs

For microinjections (Experiment 2), injection cannulas connected via tubes to two microliter syringes (10 μl, Hamilton, Switzerland) were used. Injection speed and volume were controlled by a microinjection pump (CMA 100, Schmidlein Labor + Service AG, Neuheim, CH). For the injection, the injection cannulas were put into the implanted guide cannulas and 0.3 μl of the saline or muscimol (0.15 nmol) solution was injected over 30 s. The injection cannulas remained one more minute in the brain in order to allow better diffusion. After the injections, the animal was put back into its home cage for about 15 min before it was submitted to the behavioral experiment.

Local microinjections of the GABA_A_ receptor agonist muscimol are a widely used method to induce a temporary inactivation of a brain area without affecting fibers of passage (Moser and Moser, 1998; Wilensky et al., 1999; Fendt et al., 2003). Such injections effectively block neural activity as shown by electrophysiological recordings (Krupa et al., 1999; Edeline et al., 2002; van Duuren et al., 2007; Larson et al., 2013).

### Apparatus and behavioral procedure

We used the modified open-field test which was developed to measure defensive behavior in response to alarm pheromone (Kiyokawa et al., 2006). All behavioral experiments were conducted in a rectangular arena (70 × 47 × 50 cm^3^). In one of the four edges of the arena there was a removable small hiding box (24.5 × 17.5 × 12.5 cm^3^) with an entrance hole (diameter 10 cm). The arena was located in a dimly lit room (center of the open field: ca 68 lx; background noise: 47 dB SPL).

On the first 5 days, rats were handled daily and then acclimatized to the arena (10 min). Notably, the hiding box was not placed into the arena during these acclimation sessions, and was put in the home cages for 24 h on the last day of the acclimation days.

During the experimental sessions, the animals were put into the arena which only contained a small glass vial (4.0 cm diameter; with 1 ml of the odor sample) in one corner. The rats were allowed to explore the arena with the odor for 5 min (acclimation period). Then, for the next 10 min (test period), the hiding box was put into the corner diagonally opposite of the odor sample. After the test, the tested animal was transferred into a separate cage to not transfer any odors to the yet non-tested cage mates. The arena was cleaned thoroughly with hot water and was exhausted with fresh air for about 5 min. Only after all animals from a cage were tested, the rats were again put together in the original home cage.

In Experiment 1, 13 rats were tested once with the alarm pheromone and once with a water sample (purified water). The two tests were performed on consecutive days in a balanced order.

In Experiment 2, saline (*n* = 10), or muscimol (*n* = 9) was injected into the aBNST. Then, the animals were exposed to the four odors (neck odor, female urine, alarm pheromone and fox urine). Each animal was tested with all odor samples in a balanced order on four consecutive days (Latin square design), with injections of saline or muscimol before each test.

The behavior of the animals was videotaped by a camera (Panasonic WV-CL930) fixed 30 cm above the box. For tracking the animals and further analysis of the behavior, a video tracking software was used (EthoVision XL, Noldus Information Technology, Version 8, Wageningen, NL). Head out behavior, stretched attend behavior and grooming behavior were manually scored by two experienced blinded observers (inter-observer reliability: *r*^2^ = 0.99, *p* < 0.0001). We used the following definitions (Kiyokawa et al., 2004, 2006): “Head out” is if the rat is in the hiding box and pokes the head or head and shoulders out of the entrance hole with their hind paws remaining inside the hiding box. “Conceal” is defined as the rat being entirely inside the hiding box. We defined the zone “near the stimulus” as an area of 10 cm^2^ in the edge of the stimuli. “Outside” is defined as time the rats spent in the open field.

### Histology

In Experiment 2, the brains of the rats were removed after the experiments and fixed with 4%-formaldehyde-10%-sucrose solution. On the following 2 days, sucrose concentration was increased daily by 10%. Then, 60 μm slices were cut with a cryostat (Leica CM 3050) at −22°C and Nissl-stained (1% cresylviolet). Lastly, the localization of the injection sites and brain integrity were checked with a microscope (Leica MZ 125). The injection sites were put into schematic drawings adapted from Paxinos and Watson (2014).

### Descriptive and analytical statistics

All data are expressed as means ± SEM. For statistical analysis, data were first checked for Gaussian distribution (D'Agostino and Pearson omnibus normality test). Non-Gaussian distributed data were either analyzed with non-parametric tests (Wilcoxon matched-pairs signed rank test, Mann-Whitney test). Parametric statistical tests were used if log-transformation led to Gaussian distribution. Normally-distributed data were then analyzed by *t*-test or analysis of variance (ANOVA). If appropriate, a within-subject design (repeated measure) was used. The significance level was set at *p* < 0.05 for all statistical tests.

## Results

### Experiment 1: Establishment of the modified open-field test with sprague-dawley rats

**Figure 1:**
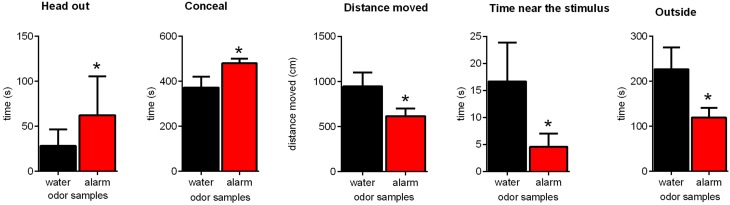
**Effects of the alarm pheromone on the behavior of Sprague-Dawley rats**. Rats were exposed to either the alarm pheromone (alarm) or water. Data are expressed as means ± SEMs. ^*^p < 0.05, paired *t*-tests.

Aim of this experiment was to establish the paradigm published by Kiyokawa et al. (2006) in Sprague-Dawley rats and to replicate these findings. Exposing the rats to the alarm pheromone in an arena induced several behavioral changes (Figure 1). Alarm pheromone exposure increased the time of head out behavior [paired *t*-test: *t*_(12)_ = 2.53, *p* = 0.03] and of conceal [*t*_(12)_ = 2.44, *p* = 0.03]. Furthermore, the time spent near the stimulus (*W*_12_ = −51.00, *p* = 0.04), the time spent outside the hiding box [*t*_(12)_ = 2.44, *p* = 0.03] and the distance moved [*t*_(12)_ = 2.29, *p* = 0.04] decreased. Stretched attend behavior and grooming behavior were only seen very occasionally and therefore excluded from further analysis.

### Experiment 2: The role of the aBNST in the alarm pheromone effects

In our second experiment, we injected either saline or muscimol into the aBNST to investigate the role of the aBNST in alarm pheromone-induced defensive behavior. In addition to the alarm pheromone, we also exposed the rats to neck odor, female rat urine and fox urine.

**Figure 2:**
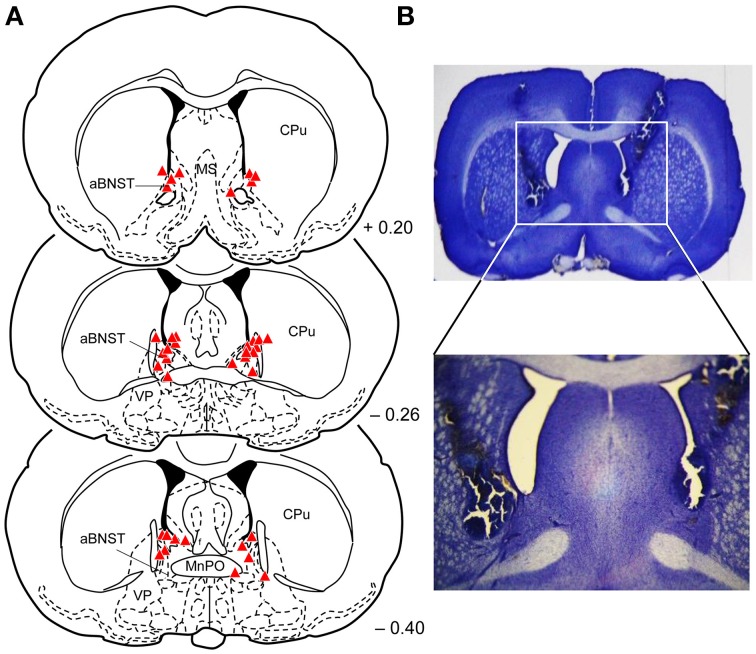
**Injection sites into the aBNST. (A)** Reconstruction of the different injection sites of saline or muscimol into the aBNST. The coronal sections were taken from the atlas of Paxinos and Watson (2014). Numbers indicate distance from bregma in mm. aBNST, anterior bed nucleus of the stria terminalis; MnPo, median preoptic nucleus; MS, medial septal nucleus; CPu, caudate putamen; LPO, lateral preoptic area; VP, ventral pallidum; f, fornix. **(B)** Photomicrographs with a representative example of aBNST injection sites.

Histological analysis of the injections sites revealed that 19 rats received bilateral injections into the aBNST (saline: *n* = 10; muscimol: *n* = 9), consisting of the anterior, dorsal and lateral divisions of the BNST (see Figure 2). Some animals had to be excluded from the analysis because of misplaced injections (*n* = 12) (lateral ventricle, medial preoptic area, caudate putamen, nucleus accumbens, lateral preoptic area, parastrial nucleus, intermediate lateral septal nucleus, medial preoptic nucleus, ventrolateral preoptic nucleus), lesions in the injection area (*n* = 5), or abnormal behavior after muscimol injections (rotation behavior; *n* = 6).

**Figure 3:**
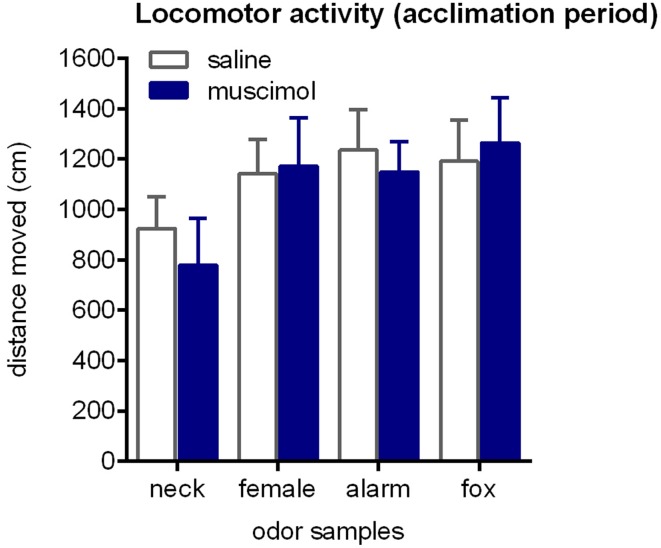
**Locomotor activity during the acclimation phase**. Rats were exposed to neck odor (neck), female urine (female), alarm pheromone (alarm), or fox urine (fox). Neither intra-aBNST injections of muscimol nor the type of odor samples affected distance traveled. Data are expressed as means ± SEMs.

First, we analyzed the distance moved in the acclimation period to check for potential effects of intra-aBNST muscimol injections on locomotor activity (Figure 3). This was clearly not the case [ANOVA: *F*_(1, 68)_ = 0.08, *p* = 0.78]. We further confirmed that locomotor activity in the acclimation phase was not affected by the type of odor [*F*_(3, 68)_ = 2.27, *p* = 0.09] and that there was no interaction between treatment and odor [*F*_(3, 68)_ = 0.19, *p* = 0.89].

**Figure 4:**
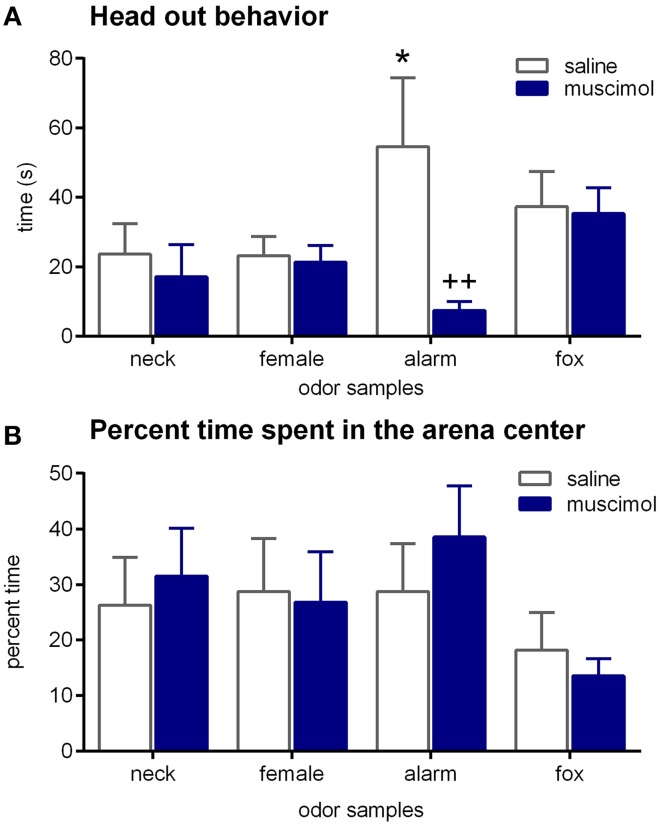
**Effects of odors and aBNST inactivation on defensive behavior**. Rats were exposed to neck odor (neck), female urine (female), alarm pheromone (alarm), or fox urine (fox). **(A)** Head out behavior, **(B)** time spent in the center, in percentage of the time spent outside. Data are expressed as means ± SEMs. ^*^p < 0.05, ^++^*p* < 0.01, comparison with saline/alarm pheromone.

During the testing period, head out behavior was the only behavior that was significantly affected by odors [Figure 4A; ANOVA: factor odor: *F*_(3, 65)_ = 2.87; *p* = 0.04]. Particularly, the alarm pheromone increased head out behavior (paired *t*-tests: *t* = 3.23, *p* < 0.01 and *t* = 2.54, *p* < 0.03; comparison with neck odor and female urine, respectively). Notable, despite statistical analysis revealed no significant effect, the time spent in the center of the arena (as percentage of time spent outside) was slightly decreased by fox urine [Figure 4B; ANOVA: factor odor: *F*_(3, 68)_ = 1.46, *p* = 0.23]. All other behaviors, such as conceal, distance moved, time near the stimulus, and outside, were not significantly affected by odors, especially alarm pheromone, in this experiment (Table 1; ANOVAs: factor odor: *Fs* < 0.61, *ps* > 0.61).

**Table 1:** **Behaviors observed in the modified open-field test**.

**Behavior**	**Treatment**	**Odor sample**				**ANOVA results**		
		**Neck**	**Female**	**Alarm**	**Fox**	**Odor**	**Treatment**	**Interaction**
Conceal [s]	Saline	463±64	470±61	484±41	537±14	*p* = 0.79	*p* = 0.23	*p* = 0.59
	Muscimol	559±9	534±16	488±70	526±20			
Distance moved [cm]	Saline	423±164	277±77	424±159	241±41	*p* = 0.95	*p* = 0.39	*p* = 0.37
	Muscimol	172±31	343±91	263±56	314±141			
Time near stimulus [s]	Saline	2.7±1.8	2.4±1.9	3.4±2.3	0.9±0.4	*p* = 0.69	*p* = 0.08	*p* = 0.91
	Muscimol	0.2±0.2	1.2±0.8	1.1±0.6	0.2±0.2			
Outside [s]	Saline	100±60	98±64	49±21	19±4	*p* = 0.61	*p* = 0.21	*p* = 0.57
	Muscimol	13±7	42±23	56±32	23±11			
Fecal boli	Saline	0.8±0.5	1.6±0.6	1.8±0.4	0.7±0.3	*p* = 0.63	*p* = 0.41	*p* = 0.51
	Muscimol	0.8±0.4	1.1±0.8	0.8±0.3	1.0±0.5			

Muscimol injections into the aBNST specifically reduced head out behavior in response to the alarm pheromone (Figure 4A). An ANOVA using treatment as between-subject factor and odor as within-subject factor revealed a significant main effect of odor [*F*_(3, 65)_ = 2.87, *p* = 0.04] and a significant interaction between treatment and odor [*F*_(3, 65)_ = 3.22, *p* = 0.03], whereas there was only a tendency for a main effect of treatment [*F*_(1, 65)_ = 3.35, *p* = 0.07]. Notably, only head out behavior during exposure to the alarm pheromone was significantly decreased by intra-aBNST muscimol injections (*post-hoc* Dunnett test: *t* = 3.41, *p* = 0.005), whereas it was not affected by muscimol injections during the exposure to other odors (*ts* < 1.04, *ps* > 0.76). It should be mentioned that bilaterally misplaced muscimol injections apparently had no effect on alarm pheromone-induced head out behavior (saline/neck: 17.0 ± 3.1 s; saline/alarm pheromone: 32.9 ± 15.3 s; muscimol/neck: 17.7 ± 3.7 s; muscimol/alarm pheromone: 28.3 ± 14.3 s). However, variability was very high in these animals and group size (*n* = 5; most misplaced injections were unilateral or into the ventricle) too small for statistical analyses.

## Discussion

In the present study, we assessed the hypothesis that the aBNST plays a crucial role in alarm pheromone-induced defensive behavior in rats. In Experiment 1, Sprague-Dawley rats showed increased head out behavior, as well as increased conceal behavior and decreased time spent outside, time spent near the stimulus, and the distance moved in response to the alarm pheromone. These results suggest that we successfully established the behavioral paradigm developed by Kiyokawa et al. (2006) in our laboratory with Sprague-Dawley rats. In Experiment 2, we showed that local muscimol injections into the aBNST lead to a blockade of head out behavior in response to the alarm pheromone. Based on these findings, we suggest that the aBNST is an important brain region for alarm pheromone-induced defensive behavior in rats.

In Experiment 1, we provided first evidence that Sprague-Dawley rats emit an alarm pheromone that induces several defensive behavior, as it was previously shown for Wistar rats (Kiyokawa et al., 2006). The present results show that the behavioral changes in response to the alarm pheromone are very similar in Sprague-Dawley and Wistar rats. Therefore, it would be plausible that Sprague-Dawley and Wistar rats share 4-methylpentanal and hexanal as active ingredients of their alarm pheromones. However, there were also some differences. In Sprague-Dawley rats, there was a clear avoidance of the alarm pheromone (Figure 1; time near stimulus), whereas Wistar rats did not avoid their alarm pheromone (Kiyokawa et al., 2006). One possible explanation might be that Sprague-Dawley rats are more sensitive to their alarm pheromone than Wistar rats. This hypothesis is supported by the findings from the forced swimming test paradigm. When rats were forced to swim in water, they released an “alarm substance” in water that decreased immobility of subsequent swimming rats (Abel and Bilitzke, 1990). The effects of this alarm substance were greater in Sprague-Dawley rats than in Wistar rats (Abel, 1992). Therefore, it is possible that a difference in sensitivity to the alarm pheromone resulted in contrasting avoidance behavior of Sprague-Dawley and Wistar rats in the modified open-field test. This is supported by studies demonstrating a greater sensitivity of Sprague-Dawley rats to other odors that can induce defensive behavior (e.g., Rosen et al., 2006; Fendt and Endres, 2008). However, it should also be noted that both Sprague-Dawley and Wistar rats were much less sensitive to the alarm substance of the other strain in the forced swimming test (Abel, 1992). Therefore, it has to be addressed by future studies if this is also the case for the alarm pheromone.

Using the modified open-field test, we assessed the role of the aBNST in defensive behavior to the alarm pheromone in our second experiment. However, in contrast to the Experiment 1, head out behavior was the only behavior that was robustly modulated by the alarm pheromone in saline-injected rats (Figure 4). Besides the difference in the repeated number of test rats underwent, one significant difference was that rats received local injections into the aBNST shortly before the behavioral tests in the Experiment 2. Although rats were acclimatized for such a procedure several times, they might still be distressed by the manipulations from the injection *per se*. Indeed, time spent outside was strongly decreased in Experiment 2 as compared to Experiment 1 (general means: 60 ± 19 s vs. 227 ± 48 s, respectively), i.e., rats tended to be in the hiding box more and spent only a short time in the open arena. This in turn means that defensive behaviors expressed in the hiding box (such as head out behavior) are more likely to be affected by exposure to alarm pheromone than behaviors expressed outside the hiding box (such as distance moved or time near the stimulus).

In Experiment 2, inactivation of the aBNST clearly decreased head out behavior in response to the alarm pheromone. These results suggest that the aBNST plays an important role in defensive behavior to the alarm pheromone and support previous studies showing an increased c-Fos expression in the aBNST when animals were exposed to alarm pheromone (Kiyokawa et al., 2005b; Kobayashi et al., 2013, 2015; Inagaki et al., 2014). The question is how the aBNST is embedded in the neural circuitry mediating defensive responses to the alarm pheromone. It was previously demonstrated that removal of the vomeronasal organ blocks the autonomic and behavioral effects of the alarm pheromone (Kiyokawa et al., 2007, 2013) indicating that the vomeronasal organ is required to detect the alarm pheromone. Then, after being transmitted to the AOB, alarm pheromone information should be transmitted to the aBNST in order to evoke defensive behaviors. Although the BNST is a part of the vomeronasal system, anatomical analyses revealed that the AOB sends its projection to the posterior part of the BNST (pBNST), rather than the aBNST (von Campenhausen and Mori, 2000). This means that the aBNST most probably receive alarm pheromone information from the vomeronasal organ via the posterior part of the BNST (pBNST). Given that the pBNST sends dense projections to the aBNST (Dong and Swanson, 2004), we hypothesize that the alarm pheromone activates the aBNST via intra-BNST connections from the pBNST. Alternatively, the medial amygdala (MeA) might be an additional candidate linkage site between the AOB and aBNST. It is known that the MeA receives direct projections from the AOB (von Campenhausen and Mori, 2000) and that the MeA sends projections to the BNST (Meurisse et al., 2009). Therefore, this anatomical evidence proposes the MeA as an additional candidate for linking between the AOB and the BNST. From the aBNST, there are several projections to the midbrain and brainstem mediating autonomic or behavioral changes. Autonomic changes are most probably mediated by projections via the paraventricular nucleus of the thalamus (Kobayashi et al., this issue) whereas behavioral changes may be mediated by direct and indirect projections to the medial hypothalamic defense system (Canteras, 2002; Canteras et al., this issue).

In contrast to the alarm pheromone, there was no robust effect of fox urine in this study. Nonetheless, we believe that the aBNST play an important role in defensive behavior to predator odor. A recent study described a significant decrease in freezing behavior in rats exposed to cat urine samples after muscimol injections into the BNST (Xu et al., 2012). In addition, it was demonstrated that the BNST is important for defensive behavior induced by exposure to trimethylthiazoline (TMT), a component of the fox anal secretion (Fendt et al., 2003, 2005). In Experiment 2, fox urine did not increase head out behavior and only slightly decrease the time spent in the center of the open field (in percentage of time spent outside) in the saline-injected rats (Figure 4), which makes it impossible to evaluate the effects of fox urine, as well as the role of the aBNST in defensive behavior to fox urine.

Taken together, we first established the modified open-field test paradigm using Sprague-Dawley rats. Second, we demonstrated that temporary inactivation of the aBNST blocks alarm pheromone-induced head out behavior, indicating that the aBNST is a crucial part of the neural circuitry involved in the defensive behavior to the alarm pheromone. Further analyses focusing on the role of the aBNST will clarify the neural mechanisms of the alarm pheromone effects.

### Conflict of interest statement

The authors declare that the research was conducted in the absence of any commercial or financial relationships that could be construed as a potential conflict of interest.
